# Effect of Eucommia water extract on gingivitis and periodontitis in experimental rats

**DOI:** 10.1186/s12903-022-02353-5

**Published:** 2022-08-05

**Authors:** Yueyue Wang, Qin Fan, Yanglong Xu, Fengjiao Zeng, Xia Liu, Dan Zhao, Lei Zhang, Guohui Bai

**Affiliations:** 1 Hospital of Stomatology, Zunyi Medical University, Zunyi, 563000 China; 2 Key Laboratory of Oral Disease Research, School of Stomatology, Zunyi Medical University, Zunyi, 563000 China; 3 Institute of Agro-Bioengineering and College of Life Sciences, The Key Laboratory of Plant Resources Conservation and Germplasm Innovation in Mountainous Region (Ministry of Education), Guizhou University, Guiyang, 550025 China; 4 Key Laboratory of Biocatalysis & Chiral Drug Synthesis of Guizhou Province, Key Laboratory of Basic Pharmacology of Ministry of Education, School of Pharmacy, Zunyi Medical University, Zunyi, 563000 China

**Keywords:** Water extracts of Eucommia, Gingivitis, Periodontitis

## Abstract

Herein, we evaluated the potential therapeutic effects of water extracts from Eucommia on periodontitis in experimental rats. We ligated the maxillary second molars of Sprague–Dawley(SD) rats with 4.0 silk threads and locally smeared *Porphyromonas gingivalis*(*P. gingivalis*) to induce gingivitis and periodontitis.After the model was successfully established, we exposed the rats to Eucommia water extracts through topical smearing and intragastric administration and evaluated the therapeutic effect of the extracts on gingivitis (for a 2 week treatment period) and periodontitis (over 4 weeks). We analyzed histopathological sections of the periodontal tissue and quantified the alveolar bone resorption levels, molecules related to periodontal oxidative stress, and periodontal inflammatory factors to assess the feasibility of Eucommia in treating gingivitis and periodontitis. We found that damage to the periodontal tissue was reduced after treatment with extracts,indicating that Eucommia has a positive effect in treating gingivitis and periodontitis in experimental rats. These findings are expected to provide the foothold for future research on secondary metabolites derived from Eucommia and guide the development of novel approaches for preventing and treating periodontal disease.

## Introduction

Periodontitis is a chronic infectious disease caused by microorganisms that destroy the periodontal tissue and cause alveolar bone resorption and loss of connective tissue. It is a major oral disease that results in tooth loss in adults and endangers general health [1]. Periodontitis has become a major concern worldwide, owing to its high incidence in both developed and developing countries. An increasing body of evidence suggests that *P. gingivalis* is a major pathogenic bacteria that play an important role in chronic and aggressive periodontitis[2–4].

Periodontitis need clinical intervention including but not limited to scaling and root planing, surgical periodontal therapy, use of microbial agents in conjunction with mechanical therapy. The commonly used control measures against periodontitis at home include mechanical approaches such as brushing, flossing and interdental brushing for adjacent tooth cleaning [5], and pharmaceutical interventions such as mouthwashes and fluorides (fluorine-containing coatings, fluorine-containing gels). Despite the current emphasis placed on oral health, not enough of it is directed to preventing periodontal disease(PD). For example, in poor or remote areas in developing countries,the correct method for brushing teeth has not been popularized, while interdental cleaning devices such as dental floss and interdental brushes have not been accepted by the general public [6]. According to the third and fourth National Chinese oral health epidemiological survey results, China's oral health literacy level and health behavior are still very low, warranting an urgent need for improvement.

Although periodontitis is not fatal, common periodontitis treatment methods can not completely recover the damaged periodontal tissue[7]. Accordingly, it is essential to identify a safe, effective and easily acceptable method for preventing and treating periodontitis.

*Eucommia ulmoides Oliv.* is a second-class rare and protected tree species in China with valuable medicinal value and a long history of medicinal use [8]. Over 138 chemical constituents with active ingredients such as lignans, cyclic allene terpenes, phenylpropanoids, flavonoids, polysaccharides, and antifungal proteins, have been identified in this species. An in-depth study of the chemical composition, coupled with assaying for the pharmacological activities and clinical application of these compounds, has revealed the extent of its potential, accounting for its broad application in medicine [9]. Current evidence suggests that the pharmacological effects of Eucommia mainly include antihypertensive activity, enhancement of immunity, regulation of blood lipids, lowering of blood sugar, prevention of osteoporosis, and anti-inflammatory, antibacterial, and anti-tumor activities. Furthermore, compounds from the plant reportedly harbor hepatobiliary and diuretic capabilities, protect nerve cells, regulate bone metabolism, and nourish kidneys. However, the use of Eucommia for the prevention and treatment of periodontal disease remains unknown, warranting further research.

The present study assessed the therapeutic effects of secondary metabolites derived from Eucommia extracts in rat models of periodontal disease. Importantly, our findings can guide future research toward the development of strategies for the prevention and treatment of periodontitis.

## Material and methods

### Animals

This animal study was approved by the Medical ethics committee of the Affiliated Stomatological Hospital of Zunyi Medical University,(Approval number:YJSKTLS-2018–2021-018A)and conducted in accordance with relevant guidelines and regulations. All methods are reported in accordance with ARRIVE guidelines.

A total of 44 male SD rats (90 g-110 g) were obtained from Changsha Tianqin Biotechnology Co.,Ltd. (SCXK2019-0013) for this study. Before the experiment, the rats were housed in a normal experimental environment for 1 week to acclimatize to that environment.

### Establishment the gingivitis model and treatment

Twenty SD rats were adaptively fed for 1 week. Add chloramphenicol, ampicillin and carboxybenzylpenicillin (the dosage is 1 g /500 ml water) to the drinking water for 1 week.,and anesthetized with an intraperitoneal injection of 0.4% phenobarbital sodium (10 mL/Kg, Yulong Seaweed Pharmaceutical Factory, Qingdao, China).Both maxillary second molars were ligated with 4–0 silk thread. The silk thread was knotted around three circles, and the silk thread was pressed into the subgingiva as much as possible. After operation, they were fed with moist feed and drank 10% sucrose water. The gingivitis model was successfully established after 2 weeks.

The 20 rats were randomly assigned into group 1 (the treatment group subjected to topical smear and intragastric administration of 800 mg/kg Eucommia water extracts once a day for 14 days) and group 2 (the control group treated with normal saline for 14 days). After 14 days of treatment, all rats were sacrificed, then oxidative stress-related molecules superoxide dismutase superoxide dismutase (SOD) and catalase (CAT) in the gingival tissues of the second molars were detected. The maxillary bones were harvested for Hematoxylin–eosin(HE) staining and observation.

### Establishment of periodontitis model and treatment

A total of 24 SD rats, adaptively fed for 1 week.Add chloramphenicol, ampicillin and carboxybenzylpenicillin (the dosage is 1 g /500 ml water) to the drinking water for 1 week.,and anesthetized with an intraperitoneal injection of 0.4% phenobarbital sodium (10 mL/Kg, Yulong Seaweed Pharmaceutical Factory, Qingdao, China).Both maxillary second molars were ligated with 4–0 silk thread. The silk thread was knotted around three circles, and the silk thread was pressed into the subgingiva as much as possible. After silk thread ligation, the bacteria were continuously inoculated at the buccal and palatal proximal, central and distal sites of the second molars, with a concentration of 1 × 10_8_ cfu/ml *P. gingivalis,* inoculated 3 times a day, with an interval of 30 min, each time 200 μL *P. gingivalis* bacterial solution was inoculated for 3 days. After operation, they were fed with moist feed and drank 10% sucrose water,and 4 rats were sacrificed after 4 weeks (based on pre-experiment).

Alveolar bone resorption was detected using X-ray and methylene blue staining, while changes in the maxillary second periodontal tissues were detected by HE staining. The remaining 20 rats were randomly assigned into two groups: group 1 (treatment group) rats underwent topical smear and intragastric administration of 800 mg/kg Eucommia water extract once a day for 28 days. Group 2 (control group) mice were treated with normal saline for 28 days. All rats were sacrificed after 28 days of treatment, followed by an HE assay to detect SOD and CAT in the gingival tissues of the second molars as well as inflammatory factors Interleukin 1 β(IL-1β)and Interferon-gamma(IFN-γ) serum.

### Plant extracts

Water extracts of Eucommia were obtained from Xi'an Tianfeng Biotechnology Co., Ltd. (Xi'an, China).

### Statistical analyses

Data were statistically analyzed using SPSS software version 18.0 and presented as means ± standard errors. An independent student's *t*-test was used to compare two groups, while a one-way analysis of variance (ANOVA) was employed for comparisons among three groups. A *p-value* < 0.05 was statistically significant.

## Results

### Establishment of the gingivitis model in experimental rats

One week after rats were smeared with *P. gingivalis*, their gingival tissues appeared pink, while their gingival margins became red and swollen. No bleeding was detected, and their teeth were not loose. At two weeks, the gingival tissues were swollen, exhibiting a dark red color and bleeding was observed, suggesting successful induction of gingivitis. Subsequently, the rats were sacrificed for sample collection.

### Quantification of oxidative stress-related molecules

**Fig. 1:**
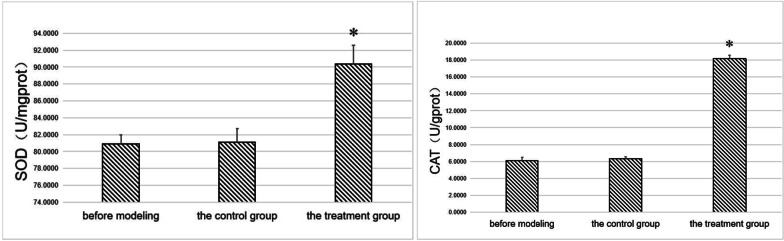
SOD and CAT in gingival tissues. *indicates a statistically significant difference during comparison with other groups (*p* < 0.05)

We subsequently quantified the levels of SOD and CAT in the gingival tissues of experimental rats according to the manufacturer's instructions of the SOD (A001-3–1) and CAT UV (A007-2–1) kits (Nanjing Jiancheng Biotechnology Research Institute). The results showed that the SOD and CAT levels in the treatment group were significantly higher than in the controls before modeling (*p* < 0.05) (Fig. 1).

### Analysis of periodontal tissues of second maxillary molars

**Fig. 2:**
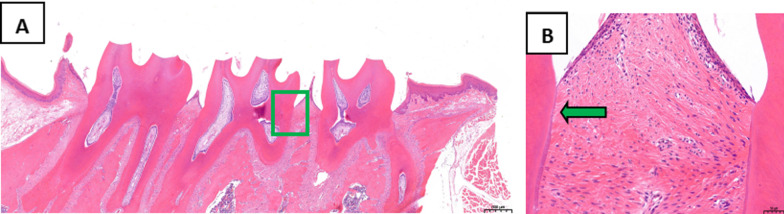
Analysis of periodontal tissues of the maxillary second molar from rats in the treatment group following HE staining, **A** Original magnification 20 × , Scale bars = 500 μm. **B** Local gums, Original magnification 200 × , Scale bars = 50 μm

**Fig. 3:**
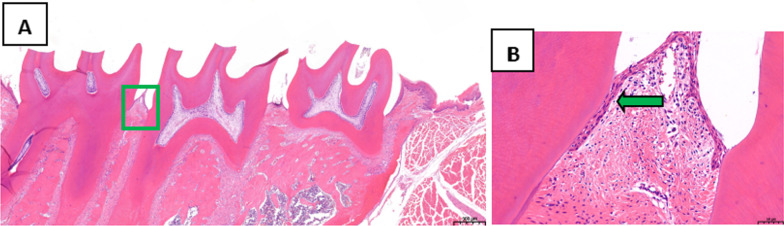
Analysis of periodontal tissues of the maxillary second molar from rats in the control group by HE staining, **A** Original magnification 20 × , Scale bars = 500 μm. **B** Local gums, Original magnification 200 × , Scale bars = 50 μm

The periodontal tissues of the second maxillary molars were analyzed by HE staining. In the treatment group, the position of junctional epithelium was restored to the enamel dentin boundary, the gingival sulcus epithelium showed no epithelial studs, and collagen fibers were formed (Fig. 2). In the control group, erosion and epithelial degeneration were observed in the gingival sulcus epithelium. The junctional epithelium proliferated and extended to the root of the teeth, while the collagen fibers exhibited hydropic degeneration (Fig. 3).

### Establishment of the periodontitis model in experimental rats

We found that the rats' gum tissues became red and swollen, exhibiting a dark red color and spontaneous bleeding at four weeks of treatment. In addition, there was a loss of adhesion, and their teeth loosened in the cheek palate direction. This preliminary finding indicated that periodontitis was successfully induced, and 4 rats were sacrificed for further analysis.

**Fig. 4:**
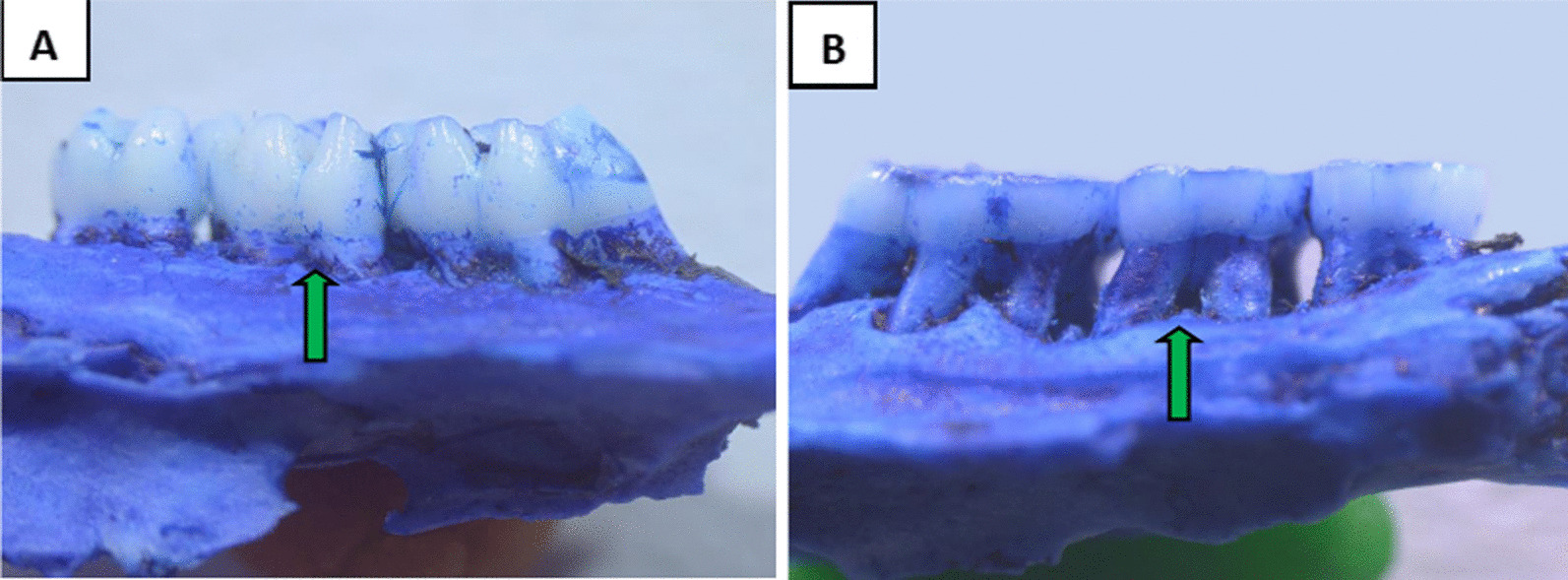
Methylene blue staining of periodontal tissues(Original magnification 20 ×). **A** The normal group. **B** the periodontitis group

**Fig. 5:**
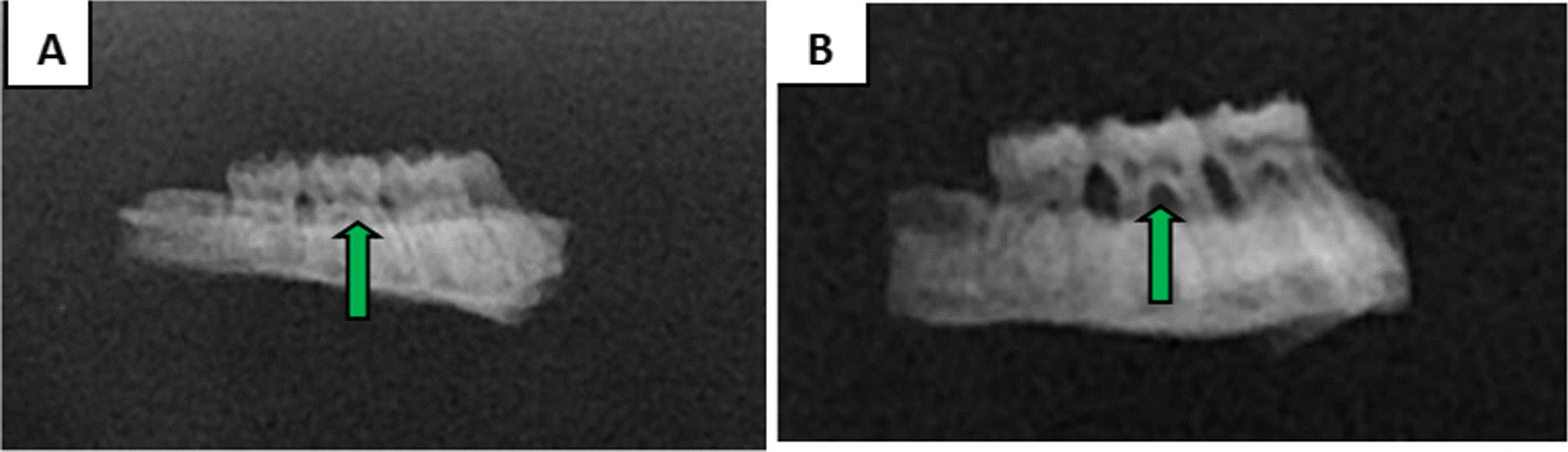
X-ray films of the periodontal tissue. **A** The normal group. **B** The periodontitis group

X-ray films and methylene blue staining showed alveolar bone resorption in the second maxillary molar in the experimental rats. The alveolar bone of normal rats wrapped around the root, which proved that periodontitis was successfully induced at four weeks of modeling (Figs. 4, 5).

### Analysis of maxillary periodontal tissues

**Fig. 6:**
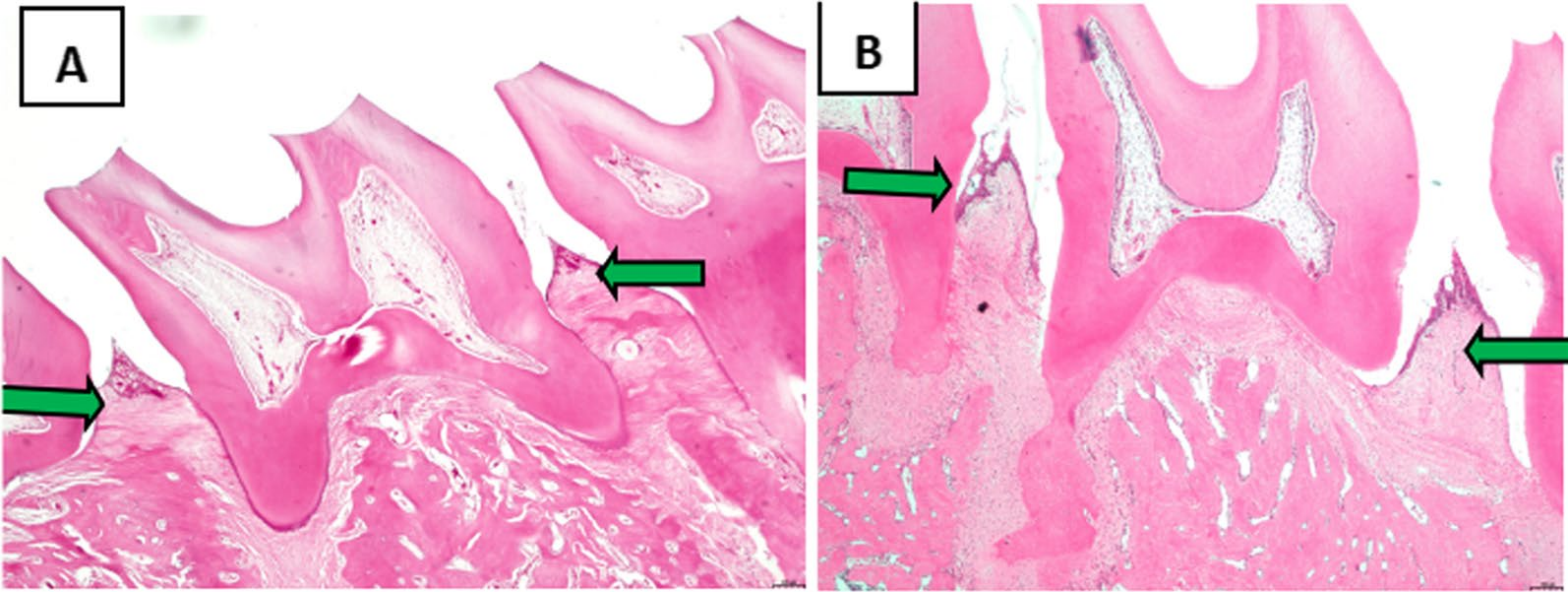
Analysis of the periodontal tissue of maxillary second molar following HE staining. Original magnification 20 × ,Scale bars = 500 μm. **A** The normal group. **B** The periodontitis group

The periodontal tissues of the second maxillary molars were analyzed following HE staining. The junctional epithelium was found at the enamel dentin boundary in normal rats, while the gingival sulcus epithelium had no epithelial studs. In addition, collagen fibers were packed into a linear arrangement..In the modeling group, erosion and epithelial degeneration occurred in the gingival sulcus epithelium. The junctional epithelium proliferated and extended to the root of the teeth. At the same time, the junctional epithelium was separated from the root surface, the periodontal pocket was formed, and significant edema was observed in the collagen fiber (Fig. 6).

### Determination of oxidative stress-related molecules

**Fig. 7:**
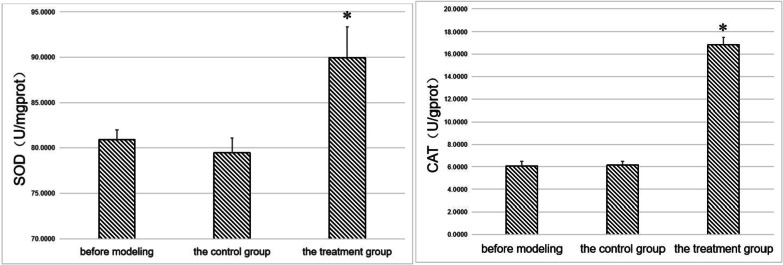
SOD and CAT in gingival tissues. *Indicates a statistically significant difference during comparison with other groups(*p* < 0.05)

Analysis of SOD and CAT levels in the gingival tissue of experimental rats showed significantly higher levels in the treatment group relative to the control group(*p* < 0.05) (Fig. 7).

### Determination of serum inflammatory factors

**Fig. 8:**
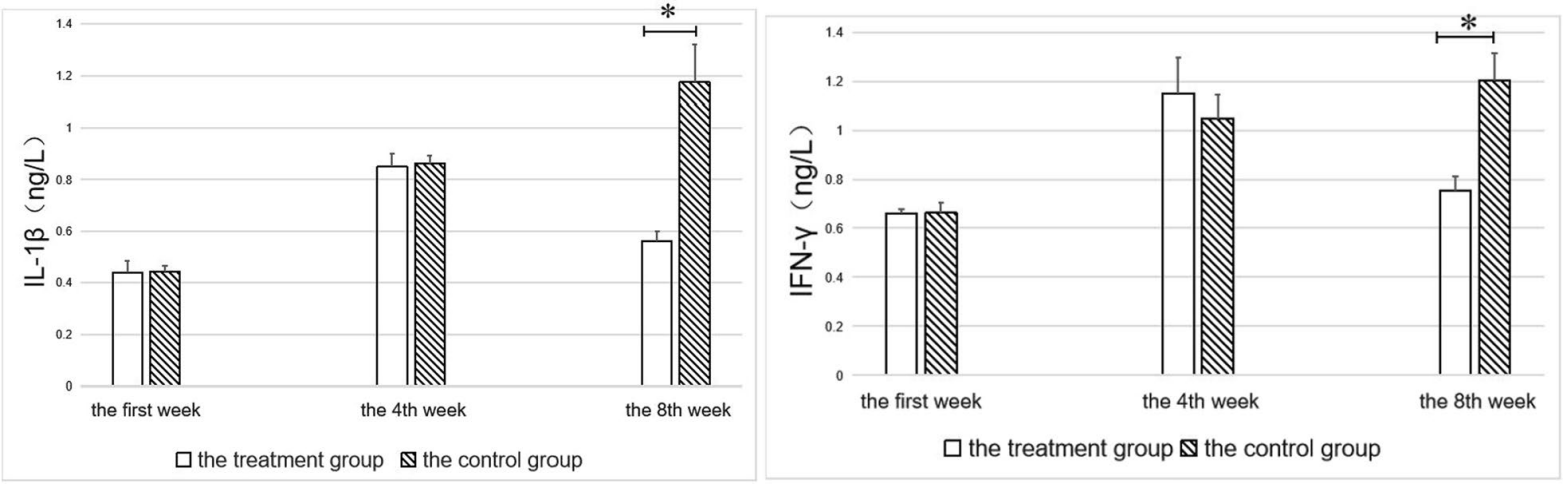
Levels of IL-1β and IFN-γ in experimental rats. *Indicates a statistically significant difference (*p* < 0.05)

We employed the ELISA technique to quantify the levels of inflammatory factors IL-1β (AndyGene, AD3023Ra) and IFN-γ (AndyGene, AD3257Ra) in rat serum. Before treatment, there was no difference between the treatment and the control groups. After treatment, the levels of inflammatory factors in the treatment group decreased, while those in the controls continued to increase. The differences between the two groups were statistically significant at week 8 (*p* < 0.05) (Fig. 8).

### Analysis of periodontal tissues of the maxillary

**Fig. 9:**
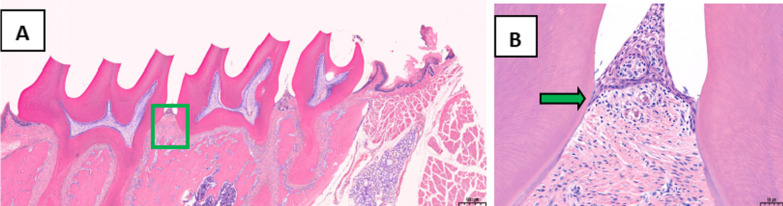
Analysis of periodontal tissues of maxillary second molar in rats from the treatment group following HE staining. **A** Original magnification 20 × , Scale bars = 500 μm. **B** Local gums, Original magnification 200 × , Scale bars = 50 μm

**Fig. 10:**
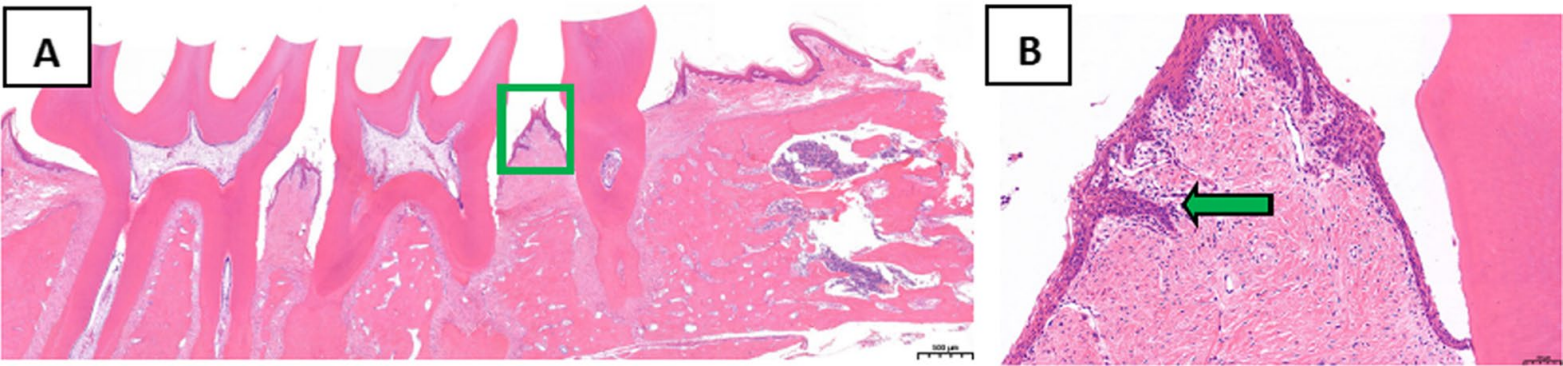
Analysis of periodontal tissues of maxillary second molar in rats from the control group by HE staining. **A** Original magnification 20 × , Scale bars = 500 μm. **B** Local gums, Original magnification 200 × , Scale bars = 50 μm

Analysis of the periodontal tissues of the second maxillary molars following HE staining indicated that in the treatment group, the position of the combined epithelium was restored to the enamel dentin boundary, the gingival sulcus epithelium had no epithelial studs, and collagen fibers were recovered (Fig. 9). In the control group, we observed erosion and epithelial degeneration in the gingival sulcus epithelium. At the same time, the junctional epithelium proliferated and extended to the root of teeth and was further separated from the root surface. Furthermore, the periodontal pocket was formed, and the collagen fiber exhibited hydropic degeneration (Fig. 10).

## Discussion

Oral diseases can occur due to external factors such as physical and chemical damage, pathogen invasion, dental and maxillofacial abnormalities and systemic diseases. Over the years, the incidence of oral diseases has significantly increased in China. The most common oral disease globally is periodontitis, which seriously affects people's health [10–12].

Overwhelming evidence substantiates that periodontal disease is also a risk factor for certain systemic diseases or conditions [13–15]. An association has been found between periodontal disease and poor blood glucose has been documented in diabetic patients [16]. Interestingly, PD has also been implicated in the occurrence and development of cardiovascular diseases [17, 18]. It is well-established that periodontal disease results from specific pathogenic bacteria [19], with clinical studies reporting that causative periodontal pathogens are found in atherosclerotic plaques. Besides, PD can cause preterm and low birth weight in infants, therefore, emphasizing the need to develop a safe, effective and easily acceptable method for its prevention and treatment [20].

To date, most research on the medicinal activity of *Eucommia ulmoides* has primarilyfocused on its ability to lower blood glucose, lipids, and blood pressure and its antioxidant and antibacterial properties [21]. However, its pharmaceutical value has not been fully harnessed [22, 23].In addition, the scope of its current clinical applications is relatively narrow, given that its leaves have not been fully incorporated into clinical applications, with the only use being for adjuvant therapy. Furthermore, few indicators are available for experimental observation, the sample size used in previous studies is relatively small and no scientific theoretical basis has hitherto been established. Indeed, research on the pharmacological mechanism of action and specific targets have been largely understudied,while in-depth, comprehensive and systematic research has not been carried out.

In this study, we assessed the therapeutic effects of Eucommia extracts on gingivitis and periodontitis. Moreover, we analyzed alveolar bone resorption levels, periodontal tissue pathological sections, and gingival tissue oxidative stress-related molecules in periodontal tissues in experimental models.. In addition, we determined changes in periodontal tissues, such as inflammatory factors. The results showed an increase in oxidative stress-related molecules SOD and CAT in the gingival tissue of the rats in the treatment group in gingivitis and periodontitis rat models treated with Eucommia extracts by topical smearing and intragastric administration. This phenomenon led to the inhibition of reactive oxygen species, thereby protecting periodontal tissues. We also found a decrease in serum pro-inflammatory factors IL-1β and IFN-γ, indicating that periodontitis inflammation was effectively controlled. HE staining of the periodontal tissues of the second maxillary molars revealed that the position of the junctional epithelium was restored to the enamel dentin boundary in the treatment group,. At the same time, the gingival sulcus epithelium had no epithelial studs, and collagen fibers recovered. In the control group, erosion and epithelial degeneration occurred in the gingival sulcus epithelium. The junctional epithelium proliferated, extended to the root of the teeth, and was further separated from the root surface. In addition, a periodontal pocket was formed, and the collagen fibers showed hydropic degeneration. These results suggest that Eucommia extracts could alleviate inflammation of the gum tissue.

Taken together, our findings indicate that Eucommia water extracts could reduce periodontal tissue inflammation and damage during gingivitis and periodontitis, indicating the therapeutic potential of this plant, but we don't know whether a component of Eucommia ulmoides has antibacterial effect. The research on the pharmacological properties of traditional Chinese medicine is extensive and profound. At present, it is an initial research. The next step is to find the monomeric compounds that play a role through mechanism research.

Summarizing the research status at home and abroad, there are still some deficiencies in the development and research of the medicinal value of Eucommia ulmoides: (1) although the basic research results on the pharmacological effects of Eucommia ulmoides in recent years are quite abundant, they have not been effectively translated into clinical application. At present, Eucommia ulmoides is mainly used in the clinical adjuvant treatment of hypertension, hyperlipidemia, hyperglycemia, tocolysis and osteoporosis. Compared with its extensive pharmacological effects, its clinical application scope is narrow, There is a serious disconnect between basic research and clinical practice; (2) At present, there are many studies on the pharmacological activities of Eucommia ulmoides extract, but due to the complex chemical components in Eucommia ulmoides, the research object is often not clear enough, the effective content is not accurate enough, and the molecular biological mechanism of pharmacological effects is not deep enough, so it is difficult to carry out comprehensive and systematic research; (3) There are few studies on Eucommia ulmoides as a clinical drug, the clinical observation indicators are not comprehensive enough, and the sample size is too small. Now it is mainly concentrated in vitro and animal experiments.

In order to speed up the excavation of the potential application value of Eucommia ulmoides in the field of biomedicine and effectively transform the basic research results into clinical applications, the author believes that the research should focus on the monomer compounds with pharmacological activities and clear structures in Eucommia ulmoides, and carry out comprehensive and systematic research on its pharmacological mechanism and action targets in vivo and in vitro, so as to speed up the transformation of basic research results into clinical applications, Improve the utilization rate of Eucommia ulmoides in the medical field, promote the sustainable development of China's great health pharmaceutical industry, and help the healthy China strategy.

## Data Availability

The data and materials used to support the findings of this study are available from the corresponding authors upon request.

## Abbreviations

*P.gingivalis*: *Porphyromonas gingivalis*
PD: Preventing periodontal disease
SD: Sprague–Dawley
SOD: Superoxide dismutase
CAT: Catalase
HE: Hematoxylin–eosin
IL-1 β: Interleukin 1 β
IFN- γ: Interferon-gamma

## References

[1] Zięba M, Chaber P, Duale K (2020). Polymeric carriers for delivery systems in the treatment of chronic periodontal disease. Polymers (Basel).

[2] Scannapieco FA, Dongari-Bagtzoglou A (2021). Dysbiosis revisited: Understanding the role of the oral microbiome in the pathogenesis of gingivitis and periodontitis: a critical assessment. J Periodontol.

[3] Blasco-Baque V, Garidou L, Pomié C (2017). periodontitis induced by Porphyromonas gingivalis drives periodontal microbiota dysbiosis and insulin resistance via an impaired adaptive immune response. Gut.

[4] Colombo APV, Tanner ACR (2019). The role of bacterial biofilms in dental caries and periodontal and peri-implant diseases: a historical perspective. J Dent Res.

[5] Mann J, Bernstein Y, Findler M (2020). Periodontal disease and its prevention, by traditional and new avenues. Exp Ther Med.

[6] Moghaddam LF, Vettore MV, Bayani A (2020). The Association of Oral Health Status, demographic characteristics and socioeconomic determinants with Oral health-related quality of life among children: a systematic review and Meta-analysis. BMC Pediatr.

[7] Mombelli A (2018). Microbial colonization of the periodontal pocket and its significance for periodontal therapy. Periodontology.

[8] Wu D, Yu D, Zhang Y (2018). Metabolite profiles, bioactivity, and HPLC fingerprint of different varieties of Eucommia ulmoides Oliv: towards the utilization of medicinal and commercial Chinese endemic tree. Molecules.

[9] Huang L, Lyu Q, Zheng W (2021). Traditional application and modern pharmacological research of Eucommia ulmoides Oliv. Chin Med.

[10] Hegde R, Awan KH (2019). Effects of periodontal disease on systemic health. Dis Mon.

[11] Kane SF (2017). The effects of oral health on systemic health. Gen Dent.

[12] Sun H, Du M, Tai B (2020). Prevalence and associated factors of periodontal conditions among 55- to 74-year-old adults in China: results from the 4th National Oral Health Survey. Clin Oral Investig.

[13] Winning L, Linden GJ (2017). Periodontitis and systemic disease: association or causality?. Curr Oral Health Rep.

[14] Bayani M, Pourali M, Keivan M (2017). Possible interaction between visfatin, periodontal infection, and other systemic diseases: a brief review of literature. Eur J Dent.

[15] Carrizales-Sepúlveda EF, Ordaz-Farias A, Vera-Pineda R (2018). Periodontal disease, systemic inflammation and the risk of cardiovascular disease. Heart Lung Circ.

[16] Nazir MA (2017). Prevalence of periodontal disease, its association with systemic diseases and prevention. Int J Health Sci.

[17] Donders HCM, Veth EO, Edens MA (2022). The effect of periodontal treatment on the reactive hyperemia index .A 1-Year Follow-Up Pilot Study. Front Cardiovasc Med.

[18] Jain P, Hassan N, Khatoon K (2021). Periodontitis and systemic disorder-an overview of relation and novel treatment modalities. Pharmaceutics.

[19] Yamashita Y, Takeshita T (2017). The oral microbiome and human health. J Oral Sci.

[20] Bartold PM, Mariotti A (2017). The future of periodontal-systemic associations: raising the standards. Curr Oral Health Rep.

[21] Zhu MQ, Sun RC (2018). Eucommia ulmoides Oliver: a potential feedstock for bioactive products. J Agric Food Chem.

[22] Zhagn XX, Sun L, Feng X (2019). Current status, problems and countermeasures of construction of standardization system of traditional Chinese medicine. China J Chin Mater Med.

[23] Jia J, Liu M, Wen Q (2019). Screening of anti-complement active ingredients from Eucommia ulmoides Oliv. branches and their metabolism in vivo based on UHPLC-Q-TOF/MS/MS. J Chromatogr B Analyt Technol Biomed Life Sci.

